# Spatial distribution and seasonal changes of mayflies (Insecta, Ephemeroptera) in a Western Balkan peat bog

**DOI:** 10.3897/zookeys.637.10359

**Published:** 2016-12-02

**Authors:** Marina Vilenica, Andreja Brigić, Mladen Kerovec, Sanja Gottstein, Ivančica Ternjej

**Affiliations:** 1 University of Zagreb, Faculty of Teacher Education, Petrinja, Croatia; 2 University of Zagreb, Faculty of Science, Department of Biology, Zagreb, Croatia

**Keywords:** Environmental factors, life cycle, mayfly assemblages, new records, peat bog

## Abstract

Peat bogs are unique wetland ecosystems of high conservation value all over the world, yet data on the macroinvertebrates (including mayfly assemblages) in these habitats are still scarce. Over the course of one growing season, mayfly assemblages were sampled each month, along with other macroinvertebrates, in the largest and oldest Croatian peat bog and an adjacent stream. In total, ten mayfly species were recorded: two species in low abundance in the peat bog, and nine species in significantly higher abundance in the stream. Low species richness and abundance in the peat bog were most likely related to the harsh environmental conditions and mayfly habitat preferences. In comparison, due to the more favourable habitat conditions, higher species richness and abundance were observed in the nearby stream. Three of the recorded species, *Caenis luctuosa* from the peat bog, and *Eurylophella karelica* and *Leptophlebia marginata* from the stream are new records for the Croatian mayfly fauna. Typical Central European life cycle patterns were confirmed for several species (e.g. *Baetis vernus*, *Nigrobaetis niger*, *Electrogena ujhelyii*), while for several others (e.g. *Habrophlebia fusca*, *Paraleptophlebia submarginata*) some discrepancies were observed. Therefore, these results provide new and valuable information on the ecology of mayflies in peat bog habitats.

## Introduction

Acidic peat bogs dominated by *Sphagnum* species occupy approximately 3% of the Earth’s land surface (Kivinen and Pakarinen 1981) and contain one-third of the world’s soil carbon (Joosten and Clarke 2002). Consequently, they play an important role in the global carbon cycle and climate change (Limpens et al. 2008, Battin et al. 2009). Peat bogs are widely distributed in boreal regions of the Northern hemisphere, but in the Western Balkans are in patches of isolated habitat (Spitzer and Danks 2006, Topić and Stančić 2006). These unique and environmentally extreme wetland ecosystems are characterized by diverse aquatic and semiaquatic habitats, high water table and acidity, low oxygen and nutrient levels (Spitzer and Danks 2006).

Peat bogs are amongst the most fragile and endangered ecosystems worldwide (Langheinrich et al. 2004) due to the climate change, agricultural activities (i.e. drainage and peat extraction) and secondary succession (Doyle 1990, Więcek et al. 2013). Even though the conservation values of bogs have been internationally recognized, these wildlife habitats are still understudied in comparison to most other freshwater habitats (Baars et al. 2014). Recent studies have shown that peat bogs are inhabited by unique macroinvertebrate assemblages, often containing rare and threatened species (Hannigan and Kelly-Quinn 2012, Drinan et al. 2013, Baars et al. 2014).

Mayflies are merolimnic insect order (i.e. with aquatic nymphal stages and terrestrial adults) with nymphs inhabiting a wide range of lotic and lentic habitats (Bauernfeind and Soldán 2012). Mayfly assemblages respond to multiple environmental factors, including water temperature (e.g. Bauernfeind and Soldán 2012), water velocity, oxygen content (e.g. Moog 2002; Bauernfeind and Soldán 2012) and pH (e.g. Fiance 1978, Petrin 2011). Mayflies are highly sensitive to habitat alterations (e.g. Zedková et al. 2015, Vilenica et al. 2016) and widely used as indicators in bio-monitoring assessments (Elliott et al. 1988, Sartori and Brittain 2015). Comprehensive data on mayfly life history traits, such as life cycles, habitat and environmental preferences are highly important for the understanding of ecosystem functioning (e.g. Brittain 1990, 1991, Raddum and Fjellheim 1993, Erba et al. 2003).

Aquatic macroinvertebrate (including mayfly) micro-distribution and ecology have primarily been studied in Northern and Central European peat bogs (e.g. Baars et al. 2014, Mieczan et al. 2015), with no comprehensive studies on mayfly assemblages in peat bogs of the Western Balkans. Thus, the aims of this study were to: 1) compare the spatial distribution of mayfly assemblages in two focal habitats: the peat bog and adjacent stream; 2) analyse environmental variables that affect the spatial distribution of mayfly assemblages and 3) determine mayfly seasonal dynamics in studied habitats.

## Methods

### Study area

**Figure 1.**
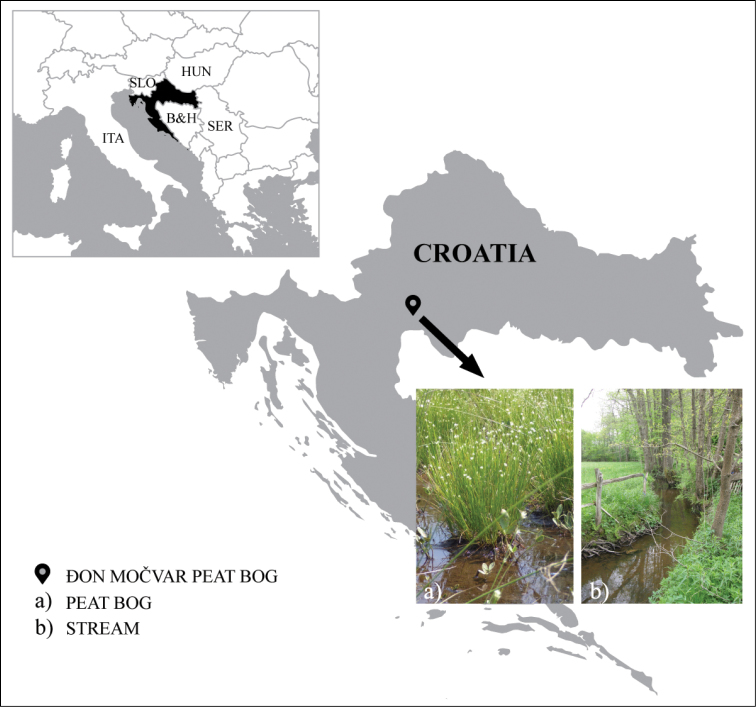
Geographical position of the Đon močvar peat bog, Croatia.

The study was conducted in the Đon močvar, one of the largest (10 ha) and oldest peat bogs in Croatia. The peat bog is located in the central part of the country (45°19'4.33"N, 15°54'32.83"E, Fig. 1) at 130 m a.s.l., under the slopes of the Petrova gora Mountain and surrounded by the Danković klada Stream. This region is characterised by a temperate humid climate (Šegota and Filipčić 2003) with an average annual temperature of 10.5 °C and an average annual precipitation of 1 050 mm (Zaninović et al. 2008).

The peat bog is a complex ecosystem, encompassing a mosaic of different habitats from open woodless *Sphagnum* spp. L. sites, deep hollows, and small ponds, to swampy areas dominated by *Rhynchospora alba* (L.) Vahl and *Phragmites australis* (Cav.) Trin. ex Steud. Abandonment of traditional land-management practices, such as mowing and grazing, has led to severe processes of succession at the peat bog. As a result, during the 20th century, the open area on the bog decreased from 40 ha to 10 ha. The peat bog and its surrounding area are protected as a Botanical Reserve and included in the NATURA 2000 network (Alegro and Šegota 2008). The Danković klada Stream is located at the peat bog edge, running through arable land and deciduous forests. It is characterized by high oscillations of water level between the seasons due to oscillations of the rainfall. The stream banks are overgrown with *Alnus glutinosa* (L.) Gaertn. and *Corylus avellana* L. Substrate composition contains mezolithal, microlithal, akal, psammal, argylal, phytal and xylal (Ternjej et al. 2015).

### Sampling and identification

Mayflies were sampled together with other aquatic macroinvertebrates at two main habitats: peat bog and stream. Within each habitat type ten replicates were collected once a month, using a benthos net (25×25 cm; mesh size = 500 µm).

In the peat bog, macroinvertebrates were collected from four different types of lentic microhabitats: lake, hollows, ditches and pools. In the stream, all major substrate types were sampled: mezolithal, microlithal, akal, psammal, argylal, phytal and xylal. The study sites differed in physico-chemical water properties, size and vegetation composition (Table 1). The samples were collected during one vegetation season, between March and November 2015.

**Table 1.** Comparison of physico-chemical water properties between the Đon močvar peat bog and adjacent stream using Mann-Whitney U test. Key: *** p<0.001, ** p<0.01, ns non-significant. The values are mean ± standard deviation.

Physico-chemical water properties	Peat bog			Stream			Mann-Whitney U test	
	mean ± SD	min	max	mean ± SD	min	max	U	p
**Water pH**	5.60 ± 0.36	5.02	6.57	6.76 ± 0.37	6.20	7.25	0.00	***
**Alkalinity (CaCO_3_ mgL^-1^)**	17.80 ± 3.50	10.00	22.50	53.75 ± 15.29	25.00	70.00	0.00	***
**Conductivity (µScm^-1^)**	37.10 ± 47.08	4.98	210.00	99.88 ± 29.81	60.00	128.00	8.00	**
**Water depth (cm)**	5.00 ± 0.56	3.93	5.60	11.84 ± 4.48	3.50	17.00	8.00	**
**Water temperature (°C)**	14.90 ± 6.81	5.20	37.00	13.22 ± 3.90	7.30	18.80	23.00	ns
**Oxygen (mgL^-1^)**	6.90 ± 2.78	1.00	11.83	7.49 ± 2.08	4.71	10.82	22.00	ns

Species were identified using e.g. Müller-Liebenau (1969), Malzacher (1984) and Bauernfeind and Humpesch (2001). Very young or damaged individuals were identified to the family level. Nomenclature follows Bauernfeind and Sóldan (2012). All voucher specimens are deposited at the Department of Biology, Faculty of Science, Zagreb, Croatia. After identification, total nymphal body length without cerci and antennae was measured using a micrometer on a dissecting stereomicroscope (Stemi 2000-C, Carl-Zeiss).

### Environmental variables

The physico-chemical water properties (water temperature, pH, dissolved oxygen concentration and conductivity) were measured at each site during each sampling date, with a multiparameter probe (WTW Multi 3430). Alkalinity (concentration of CaCO_3_ (mg/L)) was measured using Standard Analytical Procedure (APHA). Since the water was brown coloured, distrophic with low turbidity, standard methods (e.g. depth-meter) could not be applied for measuring water depth. Therefore, water depth was measured with a constructed meter.

### Data analysis

Dominance was determined according to Bick (1989). Taxa represented by > 10% of individuals are classified as eudominant, taxa with 5–10% of total abundance as dominant, taxa with 2–5% as subdominant, taxa with 1–2% as recedent and taxa with less than 1% of total share as subrecedent. In order to estimate differences in physico-chemical water properties and mayfly assemblages (number of taxa and number of individuals) between the peat bog and adjacent stream, a Mann-Whitney U test was applied. Prior to the analyses, the data were tested for normality using a Shapiro-Wilk test. These tests are based on pooled microhabitat data both from the peat bog and stream, for physico-chemical parameters and mayfly assemblages. The tests were performed using Statistica 12.0 software package (StatSoft Inc. 2013). For estimation of similarity and differences in mayfly assemblages between the peat bog and stream during the study period, a Bray-Curtis similarity index was used. Prior to analysis, the data were square root transformed. The results of hierarchical cluster analysis were superimposed on Non-metric multidimensional scaling (NMDS) plot. Samples with no mayfly records were excluded from analyses. These analyses were performed using the PRIMER v6 software package (Clarke and Warwick 2001). Life cycle patterns of eudominant and dominant mayfly species were analysed by grouping the nymphs into 1 mm body size classes. All figures were processed with Adobe Illustrator CS6.

## Results

In the peat bog, water was highly acidic, differing significantly from the stream (Table 1). Alkalinity and conductivity were three times lower in the peat bog than in the stream. Additionally, water depth was two times lower in the peat bog than in the stream. Water temperature did not differ significantly between the two habitats. However, we observed large variability of water temperature among peat bog microhabitats, particularly in shallow ditches, where summer maximums reached 37 °C. Similar variability was detected for oxygen concentration, with minimum values of only 1 mgL^-1^ in the peat bog (Table 1).

A total of ten mayfly species were recorded in the peat bog and adjacent stream (Table 2). Only two species were collected from the peat bog, *Cloeon dipterum* (Linnaeus, 1761) and *Caenis luctuosa* (Bürmeister, 1839), while in the stream nine species were recorded. *Cloeon dipterum* was the most abundant species recorded in the peat bog (Table 1), while it was the only subrecedent in the stream. *Caenis luctuosa* was found only in the peat bog with only one specimen (Table 2). In the stream, *Baetis vernus* Curtis, 1834) (22.80% of the total catch) was the most numerous species, followed by *Habrophlebia fusca* (Curtis, 1834) (20.10%) and *Electrogena ujhelyii* (Sowa, 1981) (15.50%) (Table 2).

**Table 2.** Mayfly taxa and their abundance recorded in the Đon močvar peat bog and adjacent stream. Key: * new mayfly records for the Croatian fauna.

Mayfly taxa	Peat bog	Dominance (%)	Stream	Dominance (%)
**Baetidae**				
Baetidae juvenile			106	18.40
*Baetis rhodani* (Pictet, 1843)			6	1.04
*Baetis vernus* Curtis, 1834			131	22.80
*Cloeon dipterum* (Linnaeus, 1761)	36	97.30	5	0.90
*Nigrobaetis niger* (Linnaeus, 1761)			60	10.40
**Caenidae**				
*Caenis luctuosa* (Bürmeister, 1839) *	1	2.70		
**Heptageniidae**				
*Electrogena ujhelyii* (Sowa, 1981)			89	15.50
**Ephemerellidae**				
*Eurylophella karelica* Tiensuu, 1935 *			1	0.17
**Leptophlebiidae**				
*Habrophlebia fusca* (Curtis, 1834)			119	20.10
*Leptophlebia marginata* (Linnaeus, 1767) *			1	0.17
*Paraleptophlebia submarginata* (Stephens, 1835)			57	9.91
**Species richness (S)**	**2**		**9**	
**Number of individuals (N)**	**37**		**575**	

Three species were recorded for the first time for the Croatian mayfly fauna, namely *Caenis luctuosa*, *Eurylophella karelica* Tiensuu, 1935 and *Leptophlebia marginata* (Linnaeus, 1767) (Table 2).

**Figure 2.**
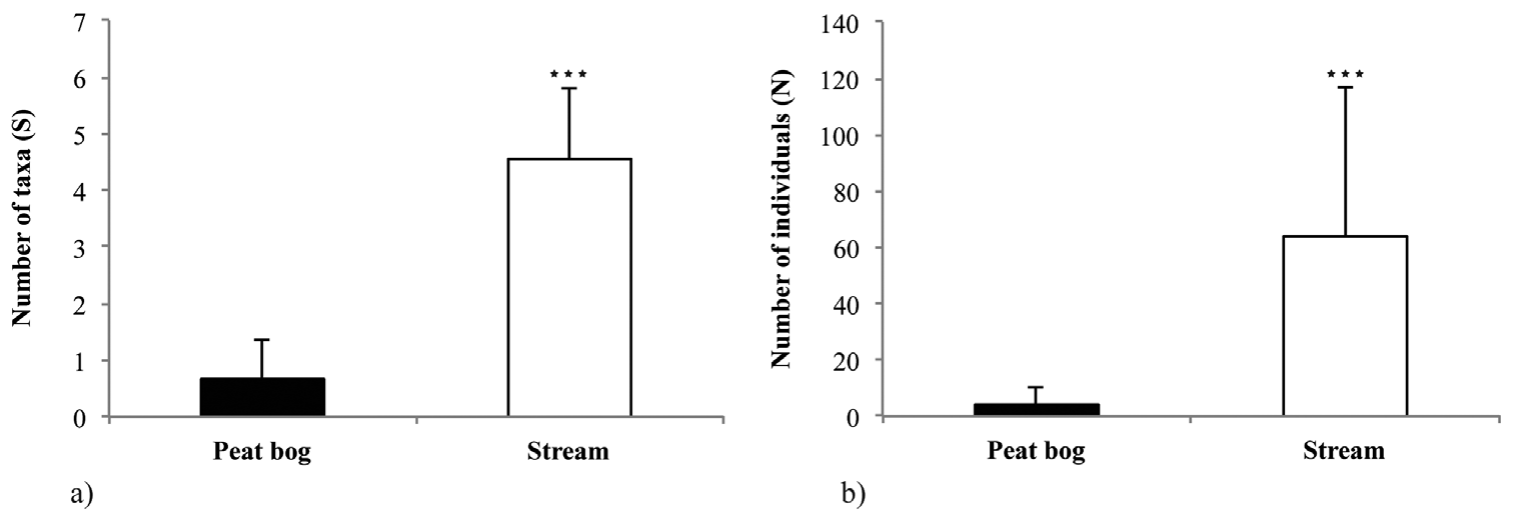
Mayfly taxa: **a** species richness (S) and **b** number of individuals (N) in the peat bog and adjacent stream (mean ± SD). The asterisk indicates significant difference between the habitats (Mann-Whitney U test, p < 0.001).

Species richness ranged from 0 to 2 in the peat bog and from 3 to 7 in the stream. It was significantly lower in the peat bog (mean ± SD, 0.66 ± 0.71; Mann-Whitney U test, U = 0.00, p < 0.001; Fig. 2a) than in the stream (4.56 ± 1.24). The number of individuals ranged from 0 to 18 in the peat bog and from 11 to 173 in the stream. There was a significant difference between the peat bog (4.11 ± 5.76) and stream (63.89 ± 52.68; U = 1.00, p < 0.001; Fig. 2b).

**Figure 3.**
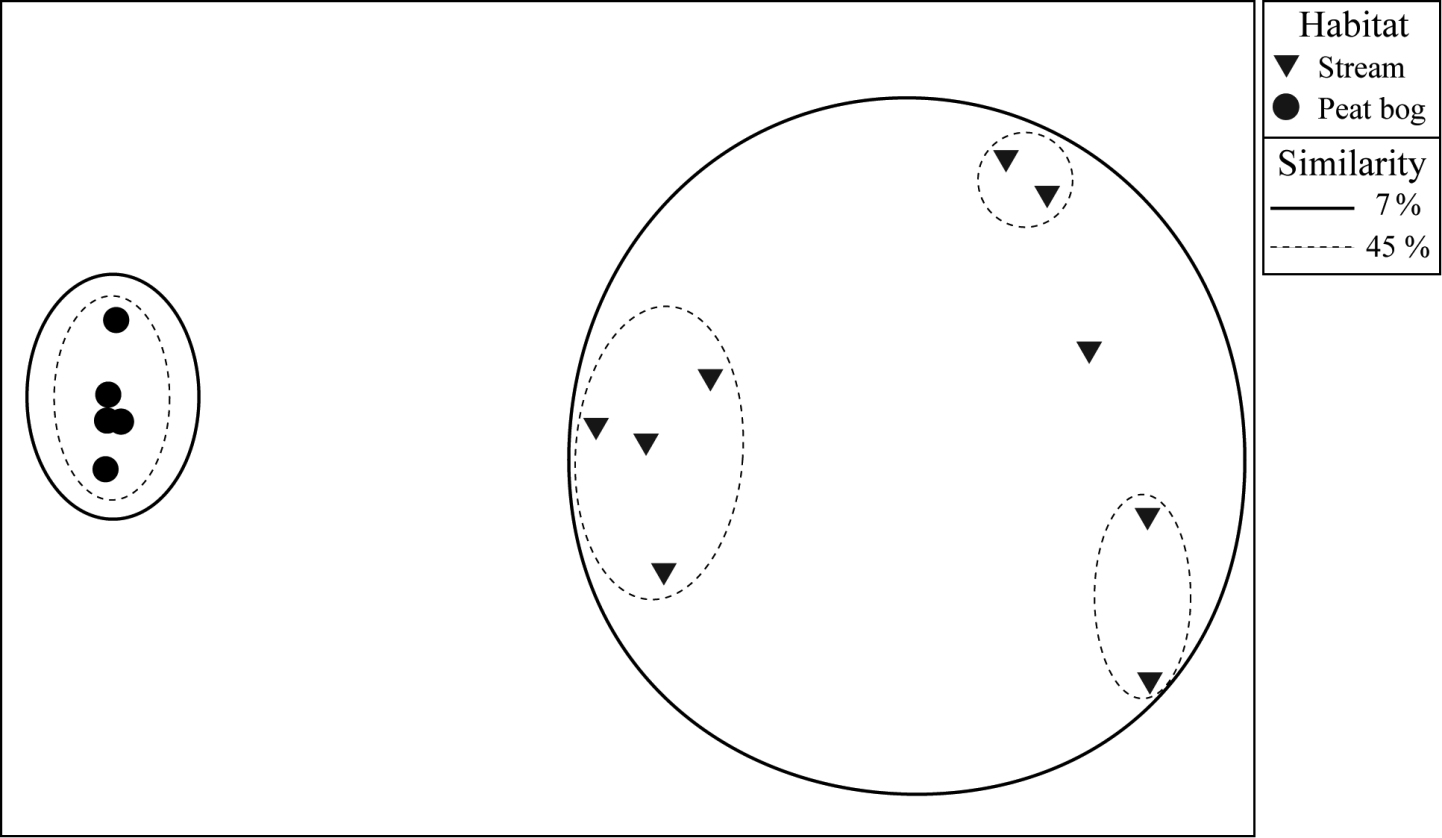
Ordination of non-metric multidimensional scaling of mayfly assemblages based on Bray-Curtis similarity coefficient (group average linking) and their square root transformed abundances, with superimposed data of hierarchical cluster analysis.

The similarity between the peat bog and stream was very low, less than 7%. Moreover, NMDS analysis showed clustering of the samples according to the habitat type: the peat bog and stream clustered separately (Fig. 3).

**Figure 4.**
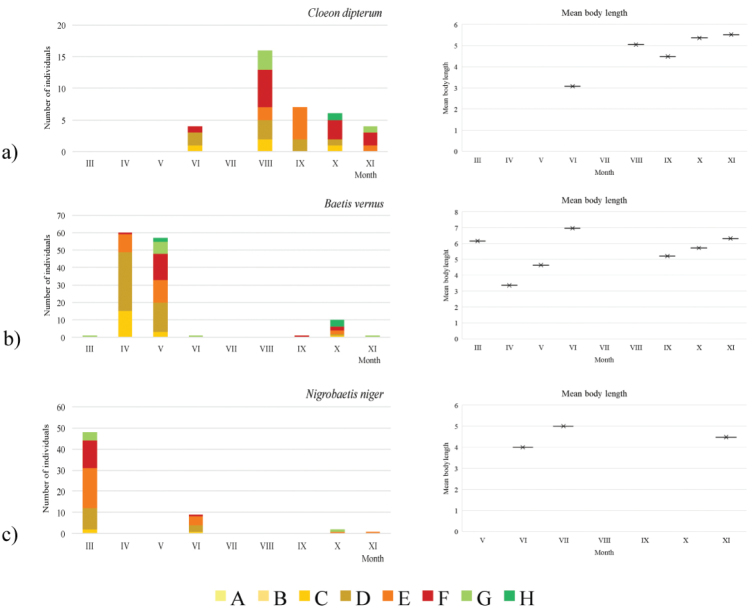
Seasonal dynamics of **a** *Cloeon dipterum* in the Đon močvar peat bog and **b** *Baetis vernus* **c** *Nigrobaetis niger* in adjacent Daković klada Stream between March and November 2015. Legend: Body length category: A = 0.00–0.99 mm; B = 1.00–1.99 mm; C = 2.00–2.99 mm; D = 3.00–3.99 mm; E = 4.00–4.99 mm; F = 5.00–5.99 mm; G = 6.00–6.99 mm; H = 7.00–7.99 mm.

In the peat bog, mature nymphs of *Cloeon dipterum* (Fig. 4a) were recorded in June and between August and November, with the highest abundance in August. The body length ranged between 2.2 and 7.04 mm.

**Figure 5.**
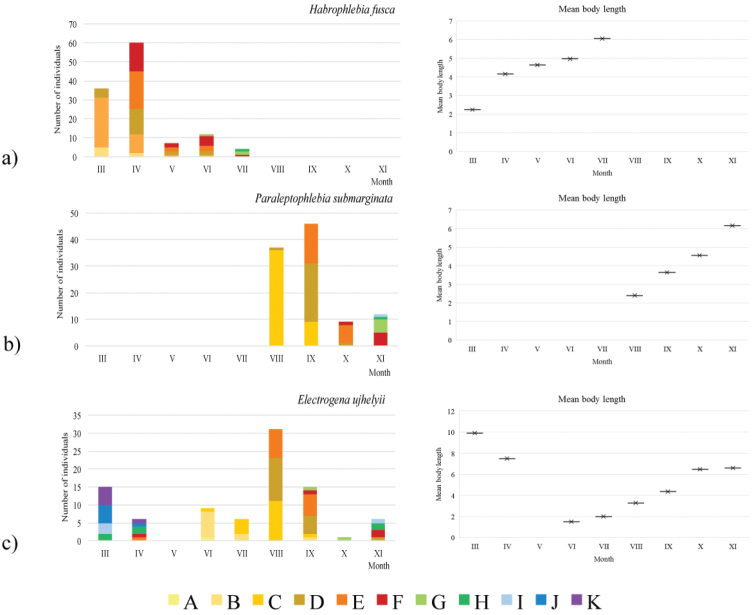
Seasonal dynamics of **a** *Habrophlebia fusca* **b** *Paraleptophlebia submarginata* **c** *Electrogena ujhelyii* in Danković klada Stream between March and November 2015. Legend: Body length category: A = 0.00–0.99 mm; B = 1.00–1.99 mm; C = 2.00–2.99 mm; D = 3.00–3.99 mm; E = 4.00–4.99 mm; F = 5.00–5.99 mm; G = 6.00–6.99 mm; H = 7.00–7.99 mm; I = 8.00–8.99 mm; J = 9.00–9.99 mm; K = 10.00–10.99 mm.

In the adjacent stream, the body length of *Baetis vernus* (Fig. 4b) ranged between 2.56 and 7.76 mm. The species was recorded between March and June and between September and November. Mature nymphs were recorded in both periods of occurrence. The body length of *Nigrobaetis niger* (Fig. 4c) ranged between 2.64 and 6.40 mm. Mature nymphs were recorded in March, June, and October. *Habrophlebia fusca* (Fig. 5a) was recorded between March and July, with mature nymphs present from April. The body length ranged between 1.60 and 7.20 mm. *Paraleptophlebia submarginata* (Fig. 5b) was recorded between August and November. The body length ranged between 2.00 and 8.16 mm, with mature nymphs present in October and November. The body length of *Electrogena ujhelyii* (Fig. 5c) ranged between 0.90 and 10.95 mm, with mature nymphs present in March, April and November.

## Discussion

This study shows that mayflies have low species richness and abundance in the peat bog, as already reported by several other studies (e.g. Bauernfeind and Moog 2000, Joniak and Domek 2006, Schartau et al. 2008). Similarly, NMDS analysis showed a low degree of similarity between the peat bog and adjacent stream. The extreme habitat conditions, such as low pH and high water temperatures were most probably the main limiting factors for mayflies. Nevertheless, two species managed to survive in such harsh environment. The eurytopic and eurythermic *Cloeon dipterum*, a typical pioneer species exhibiting traits of invasive behaviour (Bauernfeind and Sóldan 2012) was recorded at both focal habitat types (i.e. peat bog and stream). Highly tolerant species to eutrophication and high temperatures, *Caenis luctuosa*, generally inhabits lentic habitats, predominantly lakes (Bauernfeind and Sóldan 2012) and it was recorded only in the peat bog. Surprisingly, some studies show high sensitivity of this species to acidification (e.g. Joniak and Domek 2006, Schartau et al. 2008). However, the pH values at the sites in these studies were even lower (approximately 4) than in Đon močvar peat bog, which could indicate that the species is intolerable to pH values less than 5. Future studies should focus on revealing the pH tolerance of *Caenis luctuosa*.

The interplay of moderate physico-chemical water properties and a variety of microhabitats in the adjacent stream provided suitable habitat conditions for significantly higher abundances of diverse mayfly species (Bauernfeind and Sóldan 2012). When compared to some other similar streams in that area (e.g. five species recorded in Čatlan, Zeleni dol and Moštanica Streams; see in Vilenica et al. 2015), mayfly species richness recorded from the Danković klada Stream could be considered as relatively high. Mayfly assemblage composition in the stream is a consequence of the mayfly preferences for lotic habitats (e.g. Bauernfeind and Moog 2000, Bauernfeind and Humpesch 2001, Bauernfeind and Sóldan 2012), combined with neutral pH values and moderately high water temperatures. With the exception of *Electrogena ujhelyii* and *Eurylophella karelica*, whose temperature preferences are not recognized yet, all other recorded species are euritherm, with a preference for moderately warm to warm water temperatures (Buffagni et al. 2009, Buffagni et al. 2015). As water temperature was already recognized as one of the most important environmental factors influencing mayfly assemblages (e.g. Brittain 1979, Harper and Peckarsky 2006) and many authors showed that mayflies are very sensitive to low pH values (Fiance 1978, Gerhardt 1990, Petrin 2011), the results of this study are in accordance.

Mayfly adult life is very short, with the individual life span lasting approximately one day depending on the species. Thus, mayflies spend the majority of their life in the nymphal stage in aquatic habitats (Brittain and Sartori 2003, Bauernfeind and Soldán 2012). Life cycles and seasonal dynamics of most of the temperate mayfly species are well known, with about 60% of the species having univoltine, 30% multivoltine, 4% semivoltine and 3% variable life cycle types (Clifford 1982). The proportion of univoltine species in the study area is in accordance with the latter data, while the proportions of the multivoltine and variable species, show certain discrepancies. According to the literature (Clifford 1982, Bauernfeind and Soldán 2012), 60% of the recorded species were previously determined to have univoltine (e.g. *Habrophlebia fusca*, *Paraleptophlebia submarginata*), 20% multivoltine (bivoltine) (*Nigrobaetis niger*, *Cloeon dipterum*) and 20% variable (*Baetis rhodani*, *Baetis vernus*) life cycles. Semivoltine species were not recorded. Certain plasticity, i.e. discrepancies from their representative life cycle patterns were already recorded for some species in different climates and different habitats, which often results in unique patterns (e.g. Alba-Tercedor 1990, López-Rodríguez et al. 2008).

For bivoltine *Baetis vernus*, *Nigrobaetis niger* and univoltine *Electrogena ujhelyii* (Clifford 1982, Bauernfeind and Sóldan 2012, Buffagni et al. 2015), typical life cycle patterns were confirmed. For *Cloeon dipterum*, species with highly adaptive life cycles (Clifford 1982, Bauernfeind and Soldán 2012, Buffagni et al. 2015), seasonal bivoltine summer life cycle type was recorded. On the other hand, some discrepancies were observed for two Leptophlebiidae species. Some previous studies have shown that *Habrophlebia fusca* and *Paraleptophlebia submarginata* have univoltine life cycles with overwintering in the nymphal stage and mature nymphs present during the early winter season (Bauernfeind and Sóldan 2012). In the Danković klada Stream, mature nymphs of *Habrophlebia fusca* and *Paraleptophlebia submarginata* were successively recorded during the early summer and late autumn, respectively. López-Rodríguez et al. (2010) recorded similar pattern in the life cycles of some other species belonging to the same two genera. These seasonal differences in ecological niche partitioning could be related to the availability of the suitable resources in the habitat.

The current study represents an important contribution to the knowledge of the mayfly fauna in Croatia, with several new records for the country together with some records of rare species. Widely distributed, *Caenis luctuosa*, recorded only from the peat bog and *Leptophlebia marginata*, recorded only from the stream, were documented for the first time in Croatian freshwater habitats (Vilenica et al. 2015, Dekić et al. 2016). What is even more interesting, *Eurylophella karelica*, the species with a disjunct distribution, so far recorded only from Lithuania, North European Russia, Poland and Hungary (Bauernfeind and Sóldan 2012, Alain and Belfiore 2013) was also recorded for the first time (Vilenica et al. 2015, Dekić et al. 2016).

Although the Red list of Croatian mayflies does not exist yet, and none of the species is protected by the law, some recorded species are listed as rare and endangered in European Red lists (e.g. *Cloeon dipterum*, *Caenis luctuosa*, *Nigrobaetis niger*, *Habrophlebia fusca*, *Leptophlebia marginata*, *Paraleptophlebia submarginata*, *Electrogena ujhelyii*; see in e.g. Sartori and Landolt 1999, Zabric 2001). Besides newly recorded *Caenis luctuosa*, *Leptophlebia marginata and E. karlelica*, all other species are distributed both in Pannonian lowland and Dinaric Western Balkan ecoregions (ER 11 and ER 5, *sensu* Illies 1978) and in freshwater habitats of both Black Sea and Adriatic Sea Basins. *Nigrobaetis niger* and *Paraleptophlebia submarginata* were recorded in rivers and streams, *Habrophlebia fusca* in springs, rivers and streams, *Cloeon dipterum* in rivers, streams and lakes and *Electrogena ujhelyii* in springs and streams (Vilenica et al. 2015). Hence, none of these species is recorded at a critically low number of localities. However, at the localities throughout Croatia where it was previously recorded, *Nigrobaetis niger* was present in low abundances (Vilenica et al. 2015). Yet, in our study, the species was among the dominant taxa in the Danković klada Stream.

In order to evaluate more precise conservation status and threats to each of the species, additional studies are necessary at an even higher number of freshwater habitats in Croatia.

## Conclusions

With three new species records for the country, this study showed that our knowledge of the Croatian mayfly fauna is still growing. Mayfly assemblage composition and abundance in the peat bog is very impoverished and rare species can survive in such harsh environments. A number of species recorded in the adjacent stream preferably occur in lentic habitats, but can also be found in slowly flowing streams (e.g. limnophil *Electrogena ujhelyii*, *Leptophlebia marginata*, limno-rheophil *Habrophlebia fusca*, *Eurylophella karelica*; Buffagni et al. 2015). However, it seems that their dispersion to the peat bog was not possible probably due to harsh environmental conditions (low pH, high oscillations of water level and temperature).

New and rare recorded species highlight the high conservation value of the Đon močvar peat bog and adjacent stream. During the 20th century, the abandonment of traditional land-management practices, such as mowing and grazing, has led to severe processes of succession in the studied peat bog. Many of the lentic habitats have decreased in size or completely disappeared, which endangers inhabiting aquatic and terrestrial assemblages. In order to preserve unique habitats and their biodiversity in the Western Balkan region, it is of a crucial importance to protect Croatian largest peat bog from rapid successional changes.

Studies on distribution, biodiversity and ecology are particularly important for conservation planning e.g. for determining the conservation status of species and defining the factors that affect biodiversity patterns (de Silva and Medellín 2001). Thereby, knowledge of mayfly faunal composition, ecology, and seasonal dynamics could contribute to the classification and protection of the peat bog habitats in Croatia.
